# New Kids on the Block? A Bibliometric Analysis of Emerging COVID-19—Trends in Leadership Research

**DOI:** 10.1177/1548051821997406

**Published:** 2022-05

**Authors:** Robin Bauwens, Saša Batistič, Steven Kilroy, Sanne Nijs

**Affiliations:** 1 Department of Human Resource Studies, 7899 Tilburg University, Tilburg, the Netherlands

**Keywords:** leadership, COVID-19, corona, bibliometric review, science mapping

## Abstract

The COVID-19 pandemic has resulted in unprecedented challenges for society. The effects on organizations have been drastic and such tough times have demanded new organizational solutions as well as strong and new forms of organizational leadership. Leadership scholars have accelerated their research efforts in the quest to identify what is needed to lead in these uncertain times. In this paper, we adopt a bibliometric review to unravel the emerging trends in leadership research in the wake of the COVID-19 pandemic, and in doing so, identify commonalities and divergences in these themes with respect to leadership approaches and assess potential avenues for future research. The findings reveal that research on the topic has emerged along six main clusters: (1) leadership and employee health during pandemic times, (2) public leadership, (3) leadership in health care, (4) leadership and diversity, (5) educational leadership, and (6) leadership and persuasive communication. The findings reveal that across these clusters, the pandemic has sparked research on leadership approaches that deal with change and uncertainty as well as those that are less hierarchical and person centered in nature. We also notice a novel attention to context. Rather than “new kids on the block,” these trends are largely continuations of established leadership theories and approaches that see their particular importance increase in this unprecedented situation. Finally, we outline some distinct avenues for further research with regard to leadership in COVID-19 times.

## Introduction

COVID-19 does not need an introduction. Declared a global pandemic by the World Health Organization (WHO), at the moment of this writing more than 57 million people have been infected globally and its effects on financial markets and society at large appear unprecedented. Crises and unforeseen events traditionally put the spotlight on leaders, as people turn to them for much-needed answers in times of need. More than ever, we need decisive and responsive leaders, like the Jacinda Arderns, Chris Gregoires, and Leo Varadkars of this world (Al Saidi et al., 2020). However, the current pandemic also challenges leadership, as leaders need to adapt to unfamiliar and uncertain circumstances, changing requirements from followers (Sergent & Stajkovic, 2020), oftentimes also leading remotely (Bartsch et al., 2021). In response to such challenges, there has been a massive upsurge of COVID-19-related research over the last couple of months (Donthu & Gustafsson, 2020).

Leadership research seems no exception to this rule. A quick search in Google Scholar for the year 2020 with the keywords “leadership” and “corona” or “COVID” displays a staggering 23,800 hits. At this astronomical pace, it is easy to lose track of current trends and discussions, even for well-seasoned scholars. To advance and make sense of how COVID-19 drives leadership research, this paper presents a state-of-the-art emerging research at the intersection of leadership and COVID-19. The aim is to (1) identify publication trends and key debates in research on leadership and COVID-19, (2) understand what we can learn from such debates, and (3) outline potential avenues for future research. To that end, the present paper adopts a bibliometric review. Compared to qualitative and interpretative reviews, this approach allows for a more objective picture of a field’s intellectual landscape and emerging trends reviews on a bigger sample of articles (Zupic & Čater, 2015). Furthermore, bibliometric methods are increasingly adopted in reviews of leadership research to complement classical review studies (e.g., Batistič et al., 2017; Zhu et al., 2019). Our bibliometric review allows charting the territory that leadership scholars have entered to better understand and tackle the most pressing organizational and societal challenges in COVID-19 times. Identifying the most pressing contexts and the nuances of leadership scholars have sought to investigate might provide valuable insights for future leadership endeavors in tackling the COVID-19 pandemic more effectively.

## Bibliometric Analysis

**Figure 1.**
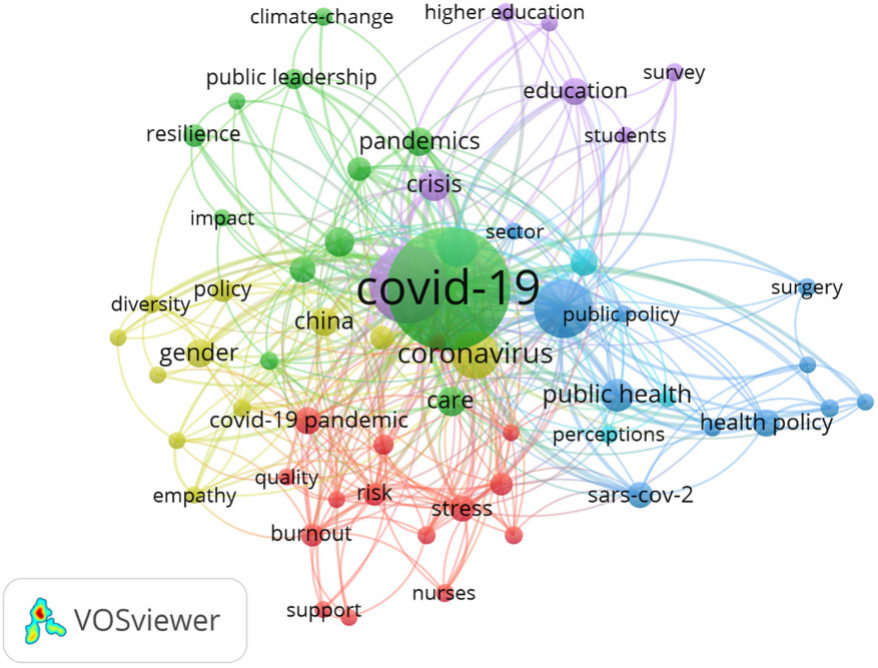
Co-word network. (Larger circles and text represent more-important keywords. The lines between the circles of keywords show that the two keywords co-occurred together. The larger the number of publications in which two terms co-occur, the stronger their relation to each other.)

This paper adopts a specific bibliometric approach, science mapping which “aims to build bibliometric maps that describe how specific disciplines, scientific domains, or research fields are conceptually, intellectually, and socially structured” (Cobo et al., 2011, p. 1382). The advantage of this technique is that it allows for a holistic overview of a particular research domain, while also minimizing reviewer subjectivity (Zupic & Čater, 2015). First, we retrieved articles from the Web of Science, the most reputable and adopted database for bibliometric techniques (Batistič et al., 2017). Based on Hossain (2020), we looked for articles that contained “leader” or “leadership” in unison with either “ncov,” “COVID-19,” “coronavirus disease,” “coronavirus pneumonia,” “coronavirus,” or “SARS-CoV-2” in their title, abstract, or keywords. Our initial search resulted in 712 records. Narrowing our search to published articles (in English) between January 1 and November 20, 2020, yielded 346 records, of which we retained 340 after duplicate screening. Contributions came from 248 different journals, most frequently from journals such as *Sustainability* (*N* = 9), *American Review of Public Administration* (*N* = 7), *Human Resource Development International* (*N* = 6), *International Nursing Review* (*N* = 5), *Journal of Professional Capital and Community* (*N* = 5), and *Leadership* (*N* = 5). Second, VOSviewer (Van Eck & Waltman, 2010) was used to subject the remaining 340 papers to co-word analysis. This technique distills the most important (key)words and how frequently they appear in combination, which provides an insight into the relatedness of research fields with a specific set of subject-related research problems shown by words and the attention paid to them by certain researchers (Braam et al., 1991). The result of this analysis can be graphed in a co-word network, which allows for identifying common clusters or themes of interest. The co-word network is depicted in Figure 1 and resulted in the identification of six main clusters. We find three large and well-connected clusters (having a frequent connection with other clusters) at the center of the network dealing with leadership and employee health during pandemic times (red), public leadership (green), leadership, and diversity (yellow). Subsequently, we observe two smaller clusters at the periphery of the network on educational leadership (purple) and health care leadership (blue). Finally, we can also distinguish the smallest cluster in the middle of the network on leadership and persuasive communication (light blue). We discuss each of these clusters in more detail below. The specific keywords within each cluster are given in the Appendix.

### Cluster 1: Leadership and Employee Health in Pandemic Times

The first identified cluster deals with employees’ well-being during pandemic times (Figure 1—red). Central research topics in this cluster are leadership in relation to burnout, stress, and mental health. Contributions come from a wide variety of journals such as *Frontiers in Psychology, Journal of Service Management*, and *Sustainability*. Most articles in this cluster deal with the challenges of leading virtually working employees. For example, Bartsch et al. (2021) found that balancing task and relational leadership behaviors helped to sustain the performance of service workers working from home. Raišiene et al. (2020) observed that in the absence of face-to-face contact with one's leader, personal leadership is important for employees working from home. Furthermore, Bhumika (2020) revealed that participative leadership accounted for a better work-life balance during lockdown. Overall, this cluster draws attention to the central role of leaders in both the prevention and mitigation of COVID-19 well-being concerns among employees.

### Cluster 2: Public Leadership

A second large cluster of research on leadership and COVID-19 deals with how leaders in different public offices have responded to the challenges posed by the pandemic (Figure 1—green). This is a very public administration-oriented cluster, with contributions from journals such as *Public Management Review, American Review of Public Administration*, and *Public Administration Review.* Central topics in this cluster are administrative and political leadership (Alam, 2021; Ansell et al., 2020), trust in public leaders (Dhanani & Franz, 2020), and public leaders’ sense-making amidst crises (Sobral et al., 2020). The key message in this cluster seems to be that there exists no single best leadership response in a crisis (Turrini et al., 2020), but that various contextual layers (e.g., policy level, county context) need to be taken into account in determining the appropriate response. This is reflected in a number of case studies of public responses, ranging from the Trump administration in the United States (Ladkin, 2020) to the Ardern administration in New Zealand (Wilson, 2020). Also, we see an increased attention to leadership approaches that are geared at dealing with uncertainty and change, such as adaptive, agile, and complex forms of leadership, approaches that are usually not addressed in public leadership discussions (Garavaglia et al., 2021; Janssen & van der Voort, 2020).

### Cluster 3: Leadership in the Health Care Sector

The third cluster of research on leadership and COVID-19 deals with leadership in health care (Figure 1—blue), a sector that has become particularly overburdened during the pandemic. The focus within this cluster is largely on nursing, reflected in outlets such as *Journal of Nursing Administration, Journal of Nursing Management*, and *British Journal of Nursing.* According to Daly et al. (2020), the COVID-19 pandemic has highlighted fundamental gaps in nursing leadership. In particular, having a vision, challenging the status quo, fostering professional development, and defending the interests of nurses seem to be blind spots. This void seems to have stirred calls to rethink nursing leadership. Indeed, leadership approaches that are more ethical (Markey et al., 2021), participatory (Allameh et al., 2020), and pay more attention to communication, intellectual, and emotional connectivity (Stephens et al., 2020) seem ever more pertinent and timely. Furthermore, scholars in this cluster also draw attention to how the pandemic has forced the other nurses and health care staff (Newell, 2020) as well as professional associations (Huang et al., 2020) to emerge as leaders in this vacuum.

### Cluster 4: Leadership and Diversity

The key topic in the fourth cluster is how the COVID-19 pandemic has highlighted or rather accentuated existing inequalities, thereby requiring a different leadership response (Figure 1—yellow). Not surprisingly, inclusive leadership is a central approach in this cluster (Ahmed et al., 2020; Kalina, 2020). The majority of articles in this cluster address gendered dimensions of leadership, which is reflected in quite a number of contributions from *Gender in Management*. For example, Rubenstein et al. (2020) argued that the COVID-19 has been especially catastrophic for women, who have suffered more in terms of economic opportunities and additional care duties. This makes gender equality in leadership positions all the more pressing to ensure adequate representation when decisions are made that affect women. Several contributions within this cluster also hail the notable positive effects of female leadership during the pandemic (Gedro et al., 2020; Sergent & Stajkovic, 2020) or advance more hybrid, androgynous approaches to leadership (Blake-Beard et al., 2020).

### Cluster 5: Educational Leadership

With the closure of schools and turn to online teaching, it should come as no surprise that COVID-19 has had a clear impact on education, resulting in a separate cluster on educational leadership (Figure 1—purple). Just like in the cluster on health care leadership, shared and emergent leadership are prevalent themes (Fernandez & Shaw, 2020; Harris, 2020) since such leaders allow for a “greater degree of agility, innovation, and collaboration” (Fernandez & Shaw, 2020, p. 39). Another prevalent theme, reminiscent of more paradox and complexity approaches, is that of the pandemic requiring leaders to manage different tensions simultaneously; for example, between autonomy and accountability or well-being and workload (Netolicky, 2020; Stone-Johnson & Weiner, 2020).

### Cluster 6: Leadership and Persuasive Communication

In the center-right of the co-word network, one finds a cluster dealing with the communication and perception of leaders (Figure 1—light blue). Its small size might be an indication that this is a more emergent cluster in the network, although it is closely intertwined with the public leadership (cluster 2), education (cluster 5), and health care clusters (cluster 3). Again, contributions come from a wide variety of journals such as *Human Resource Development International*, *Psychological Trauma*, and *Nature Human Behavior*. This cluster deals primarily with leader's communicative behavior, with a particular emphasis on social media and videoconferencing to reach out to followers (Frei-Landau, 2020). For example, McGuire et al. (2020) observed that consistent and empathic communication is key to inspire confidence and social solidarity in a crisis, while Van Bavel et al. (2020) warn against leaders expressing too much optimism in their communication these times.

## Discussion and Conclusions: New Kids on the Block?

Looking across the identified clusters, we identify some trends and in so doing unpack possibilities for future research. To a large extent, these trends are not necessarily “new kids on the block” and do not represent a radical departure from traditional leadership theories. The research questions and leader approaches across these different clusters fit within ongoing leadership debates, such as those on leadership and technology (Avolio et al., 2014), diversity and inclusion (Carmeli et al., 2010), or communication (De Vries et al., 2010). They even date back to the earliest studies of leadership from the Ohio and Michigan studies, which advocate the importance of task (productivity) and relationship (employee) behaviors. Therefore, based on our results, the trends highlighted below follow some of the calls raised by recent review pieces for more adaptive, shared, and contextual approaches (Dinh et al., 2014; Zhu et al., 2019). However, they also extend these trends by seeing their importance increased in this pandemic in light of uncertain events, leadership voids, and the differential effects of COVID-19 on particular sectors.

### Trend 1: Uncertain Events Trigger Adaptive and Agile Responses

Speaking almost prophetic words, Parker et al. (2015, p. 119) noted 5 years ago that “traditional leaders believe that more control results in more order. Unfortunately, this conventional view does not really help in the uncertain real world … unforeseen events can befall the best of plans in an instant”. Consequentially, we find that COVID-19 seems to have drawn the attention toward leadership approaches that deal with change, uncertainty, and complex challenges, such as adaptive and agile leadership. We find them, for example, in clusters on public leadership (cluster 2—e.g., Garavaglia et al., 2021; Janssen and van der Voort, 2020) and educational leadership (cluster 5—Fernandez and Shaw, 2020). Situated within contingency theory approaches (Dinh et al., 2014), adaptive leadership is reactive in “changing behavior in appropriate ways as the situation changes” (Yukl & Mahsud, 2010, p. 81), while agile leaders are more proactive by keeping in mind different scenarios, daring to experiment, dealing with paradox, and learning from failure (Parker et al., 2015). Given these types of leaders are better at anticipating future needs or events and upholding goals in disruptive situations (e.g., sudden closures of business, sanitary precautions, turn to telework), we foresee that more research efforts will emerge in this fruitful area in the coming months or years.

### Trend 2: Filling the Leadership Gaps the Pandemic has Highlighted

A second research trend has been the turn toward leadership approaches that are less hierarchical in nature, such as shared, participative, and emergent leadership. COVID-19 seems to have highlighted particular gaps or even absence of leadership and as a result, others have emerged into this void. This is a theme that we see addressed in the health care sector, with regular nurses surfacing not only in leadership roles (cluster 3—Huang et al., 2020; Newell, 2020), but also in public leadership where we see women and experts now taking the wheel (cluster 2—Al Saidi et al., 2020; Turrini et al., 2020; Wilson, 2020). These so-called “outsiders” are particularly interesting because their leadership position is often based on criteria that challenge many existing stereotypes of leaders such as being male, at the top of the organizational hierarchy, and possessing charismatic leadership qualities as the route to leader effectiveness (O’Riordan et al., 2019). Indeed, leadership is now attributed to those who have the competencies (Sergent & Stajkovic, 2020) or communication skills (cf., cluster 6)—an area where women leaders seem to excel (cluster 4—Blake-Beard et al., 2020; Dirani et al., 2020). Hence, we expect this crisis to have a profound impact on research on emergent and outside leaders, as well as the criteria that foster their emergence. However, complex situations such as pandemics might also require leadership responses that outstrip individuals (Pearce & Wassenaar, 2015), in favor of approaches such as shared and participative leadership that seeks to split the burden of crisis decision making. This was widely apparent, for example, in health care (cluster 3—Allameh et al., 2020) and education (cluster 5—Fernandez and Shaw, 2020; Harris, 2020). Hence, as argued by Ladkin (2020—cluster 2), the current pandemic could be the black swan event that cools our romantic relationship with the heroic leader as a single charismatic individual.

### Trend 3: Context Matters (Again)

That leadership does not occur in a vacuum is increasingly recognized and a recurrent theme in overview works (e.g., Batistič et al., 2017; Dinh et al., 2014; Zhu et al., 2019). The current pandemic seems to underscore that message since three of the six identified clusters deal with sectoral contexts, where COVID-19 has had severe implications for work: public (cluster 2), health care (cluster 3), and educational leadership (cluster 5). Despite some similarities (cf., trends 1 and 2), particular leaders’ approaches are more dominant in certain clusters. In the public sector, more agile and adaptive leadership seems necessary to be more responsive in crises when confronted with unwieldy organizational structures (cluster 2—e.g. Garavaglia et al., 2021; Janssen and van der Voort, 2020). In the health care sector, participative and shared leadership appear as apt responses to share some of the responsibilities and decision-making in light of patient rushes (cluster 3—e.g., Huang et al., 2020; Newell, 2020). In the education sector, the combination of unwieldy structures and responsiveness to students seems to warrant a hybrid combination of both (cluster 5—e.g., Fernandez and Shaw, 2020). Hence, while the current pandemic might be called a “great equalizer,” research on leadership and COVID-19 seems to demonstrate more than ever that context affects “the emergence and manifestation of leadership processes” (Dinh et al., 2014, p. 41). From a practical point of view, such contextual approaches are also instrumental to provide more evidence-based recommendations to pandemic-affected sectors and organizations.

### Future Directions

The first avenue is to ask who is missing in these recent contextual approaches. We see little or no attention to employees working in logistics, restaurants, events, or flexible employment. These sectors have also suffered from COVID-19—think about closures or upsurges in online shopping, but do not necessarily have a strong academic tradition built around them. This is also reflected in the position of some of these clusters. Studies of leadership and COVID-19 in health care and education we find more at the periphery of the network, in their own journals, without strong connections with other research fields. Both for paying attention to “forgotten” contexts and bringing education and health care closer to the center of the network, we see a key role for specialized leadership journals as “boundary spanners” of these different traditions, with the exception of *Journal of Leadership Studies, Leadership,* and *Journal of Applied Psychology*, contributions of such journals have been limited. The second avenue is to further assess how COVID-19 is driving leadership in virtual and remote work. While we do see some studies discussing this matter in the first cluster (e.g., Bartsch et al., 2021; Raišienė et al., 2020) and authors such as Dirani et al. (2020) stress leaders’ role as technology enablers, we expected this to be a more recurrent theme across clusters. Further development of this line of research is important to bring COVID-19 research in line with e-leadership theory (Avolio et al., 2014), especially since remote work is likely to remain important after the pandemic. The third avenue concerns leader communication. This pandemic underscores the importance of leaders to connect and reach out to followers. As argued by Dirani et al. (2020, p. 389), “leaders need to provide correct and the most updated information…[and] adopt innovative ways to feed information in moderation to employees to reduce anxiety and fear.” The presence of a small communication cluster in our co-word network suggests that this presents a promising avenue of exploration, which can undoubtedly benefit from the large leadership expertise in this area (De Vries et al., 2010).

This study is limited in at least two respects. First, we restricted our analyses to the Web of Science. Although this is the most adopted database for bibliometric reviews, future research could crosscheck with other databases such as Scopus, Ebsco, and PubMed. Second, while this study presents an early attempt to unravel the intellectual structure, the current pandemic is still ongoing and only its short-term effects are visible. Therefore, the true influence of COVID-19 on leadership research will need to be re-evaluated in the coming years. Nevertheless, one thing is sure: despite the severity of the current situation, it presents a ripe and exciting time to study leadership.

## Appendix

Table 1 provides keywords per cluster.

**Table 1.** Specific Keywords Within Each Cluster.

	Keyword	Occurrences	Weight
*Cluster 1: Leadership and employee health in pandemic times*	Anxiety	5	10
	Behavior	4	19
	Burnout	7	22
	Covid-19 pandemic	10	13
	Health care	7	19
	Influenza	4	11
	Lessons	4	16
	Mental health	4	6
	Nurses	5	9
	Quality	4	10
	Risk	7	19
	SARS	5	16
	Stress	9	32
	Support	4	6
	Work	6	21
*Cluster 2: Public leadership*	Care	12	33
	Climate change	5	4
	Covid-19	170	295
	Crisis management	12	29
	Governance	8	26
	Impact	4	6
	Pandemics	11	28
	Performance	9	25
	Public leadership	6	15
	Resilience	7	9
	Satisfaction	5	14
	Trust	4	8
*Cluster 3: Leadership in the health care sector*	Advocacy	4	13
	Global health	4	12
	Health policy	10	18
	Nursing leadership	5	13
	Pandemic	43	103
	Personal protective equipment	4	11
	Public health	14	33
	Public policy	4	16
	SARS-CoV-2	9	23
	Sector	4	10
	Surgery	5	7
*Cluster 4: Leadership and diversity*	China	11	27
	Coronavirus	28	70
	Culture	4	14
	Diversity	5	14
	Empathy	4	14
	Gender	11	23
	Health	8	19
	Model	5	17
	New Zealand	4	7
	Policy	7	13
	Social distancing	4	5
*Cluster 5: Educational leadership*	Challenges	4	8
	Crisis	13	44
	Education	10	23
	Higher education	5	10
	Leadership	73	159
	Students	4	10
	Survey	5	8
*Cluster 6: Leadership and persuasive communication*	Communication	10	32
	Experience	4	10
	Management	21	65
	Perceptions	5	8

*Note.* The weight column shows the total strength of the links of a word with other words. In this case, the weight indicates the total strength of the co-occurrence links of a given word with other words. In general, the higher the weight, the more important a word is to the network.
